# Understanding the Geographical Spread of COVID-19 in Relation with Goods Regional Routes and Governmental Decrees: The Lombardy Region Case Study

**DOI:** 10.1007/s42979-021-00597-6

**Published:** 2021-04-09

**Authors:** Davide Tosi, Marco Chiappa

**Affiliations:** 1 Department of Theoretical and Applied Science, Università degli Studi dell’Insubria, Via O. Rossi, Varese, Italy; 2 Università IULM, Componente dell’Osservatorio su Comunicazione Pubblica, Public Branding e Trasformazione Digitale, Via Carlo Bo, 1, 20143 Milan, MI Italy

**Keywords:** Data analytics, Big data, COVID-19, Good and logistic hubs

## Abstract

In this paper, a preliminary data analytics study on the spread of COVID-19 in Italy and Lombardy Region is reported to understand how the COVID-19 spread from a geographical point-of-view. Understanding the rules followed by the virus in its spread in Italy and Lombardy Region is fundamental to act properly to reduce and isolate the contagious promptly. The paper investigates the possible correlation between the goods and transport routes and the citizen travels with the spread of the COVID-19 from a geographical perspective. Moreover, in the paper, an empirical discussion is reported on how the actions and decrees, which have been imposed by the Italian Government over time, impacted actually on the spread of COVID-19. This discussion can highlight important aspects that can be taken into account in future pandemic waves, to better balance the policies and actions imposed by the Government and by the Regions.

## Introduction

The uncontrolled and unpredicted spread of COVID-19 [1, 2] in Italy [3] and in particular in Lombardy Region (RL) [4] at the end of February 2020 raises the big question: “why we were not able to predict and anticipate the wave of COVID-19 in Italy?”.

In this paper, we start from the previous authors experiences in big data smart-cities, correlation studies [5–8], and COVID-19 predictions [9, 10] to try to answer this question by studying the correlation between the COVID-19 geographical diffusion over time, with several independent variables, such as:

- the main logistics routes for goods and foods transportation in Lombardy Region;
- the people movements and travels for citizens in Lombardy Region.

Moreover, the paper discusses the legislation introduced by the Italian Government to understand whether the introduced restrictions had a real impact on the COVID-19 spread.

## A Correlation Study on Goods and Foods Logistics Routes and Citizen Travels

In this section, we study whether a correlation exists between the dependent variable COVID-19 spread with the two independent variables: logistics transport and also citizen travel in RL.

**Fig. 1:**
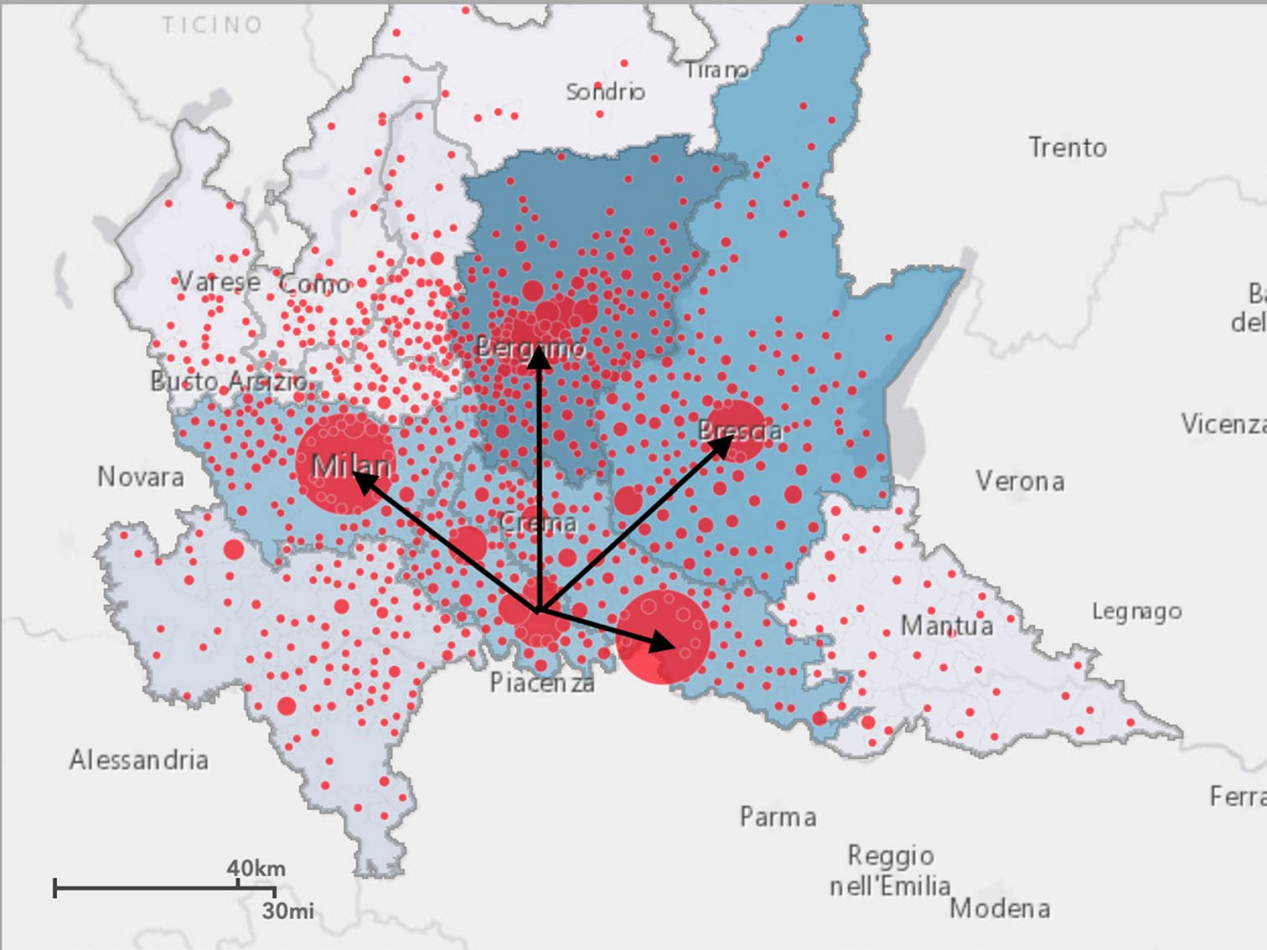
Geographical routes and spread of COVID-19 at Lombardy Region (March 11, 2020)

To this end, we started analyzing the daily geographical spread of COVID-19 in RL by studying the entire open dataset available at [11] for the 12 Provinces we have in RL: Bergamo (BG), Brescia (BS), Como (CO), Cremona (CR), Lecco (LC), Lodi (LO), Mantua (MN), Milan (MI), Monza (MB), Pavia (PV), Sondrio (SO), and Varese (VA) (see Fig. 1. for a geographical picture of the COVID-19 spread at March 11, 2020.) Of course, the main focus was in the first outbreak detected in Italy, and specifically in the town: Codogno (Lodi, RL). Then, we downloaded the whole dataset about annual goods and food movements between all the Provinces in RL (the dataset computes the numbers of all the good and food vehicles moving daily across all the routes and Provinces at Lombardy Region.) Moreover, we downloaded the whole dataset relating to daily people movements and travels between all the Provinces in RL. The two datasets are available at [11]. With these two datasets, we were able to compute Origin/Destination matrixes (O/D) to understand the connection of all Provinces from a logistics and also travel points-of-view, respectively.

**Fig. 2:**
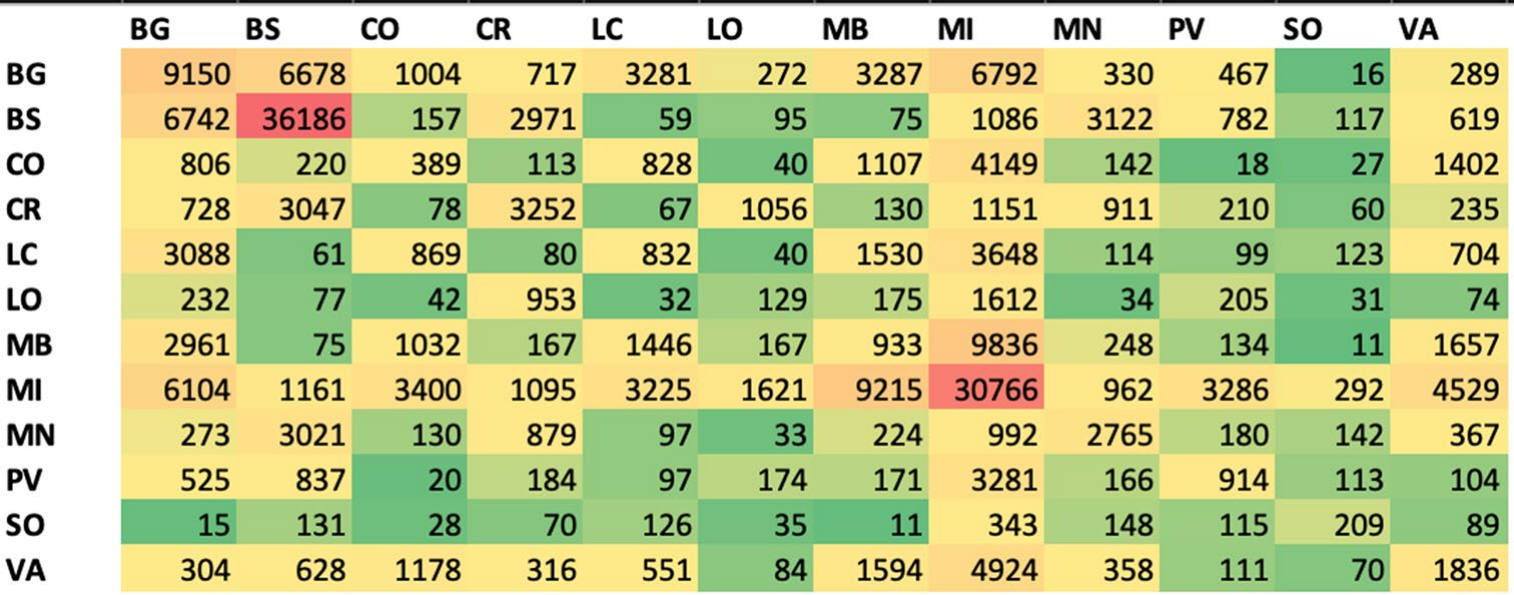
O/D matrix for goods and foods transportation for all the Provinces in Lombardy Region (number of vehicles/year)

**Fig. 3:**
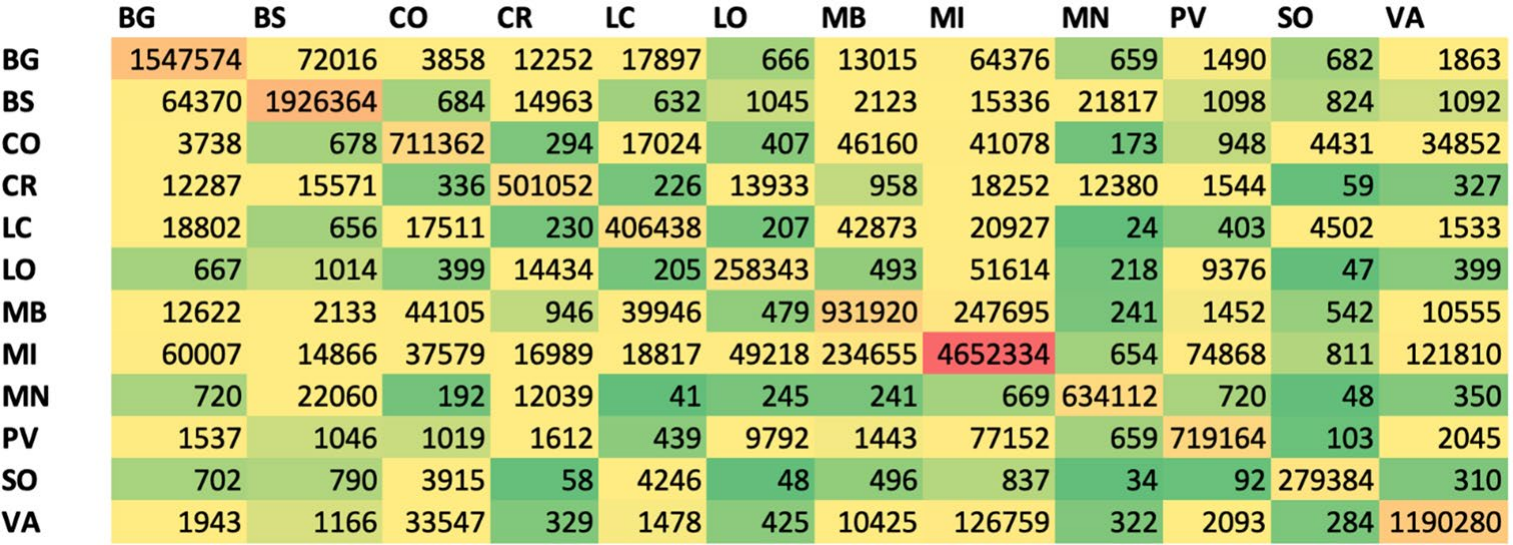
O/D matrix for population intra/inter-travels for all the Provinces in Lombardy Region (number of travels/year. Last available dataset: 2015)

The O/D matrixes are shown in Figs. 2 and 3. Figure 2 shows the O/D matrix related to the logistics transportation of goods and foods in Lombardy Region. Figure 3 shows the O/D matrix relating to the people movements in RL. The matrixes couple the different Provinces in the form ProvX–ProvY and shows, for each pair, the cumulative number of vehicles and people moving in this ProvX–ProvY connection, respectively.

Moreover, we highlighted with a chromatic scale each pair, ranging from green (low density) to red (high density) to have a quick graphical perception of the most congested and traveled connections.

As for goods and foods transport, for example, Fig. 2 shows that Milano and Brescia have congested logistics situations inside their Province (red box), while the O/D pair less congested is the one between Monza Brianza and Sondrio (green box). Focusing on the first COVID-19 outbreak in Italy at Codogno (Lodi—RL), the matrix in Fig. 2 shows that the pair LO–MI (1621 vehic./day), LO–CR (1056 vehic./day), LO–BG (272 vehic./day), and LO–PV (174 vehic./day) are the most congested connections. This is not true in the opposite direction: MI–LO is one of the less congested connections for Milan, the same for BG–LO, while CR–LO and PV–LO are very important logistics connections.

**Fig. 4:**
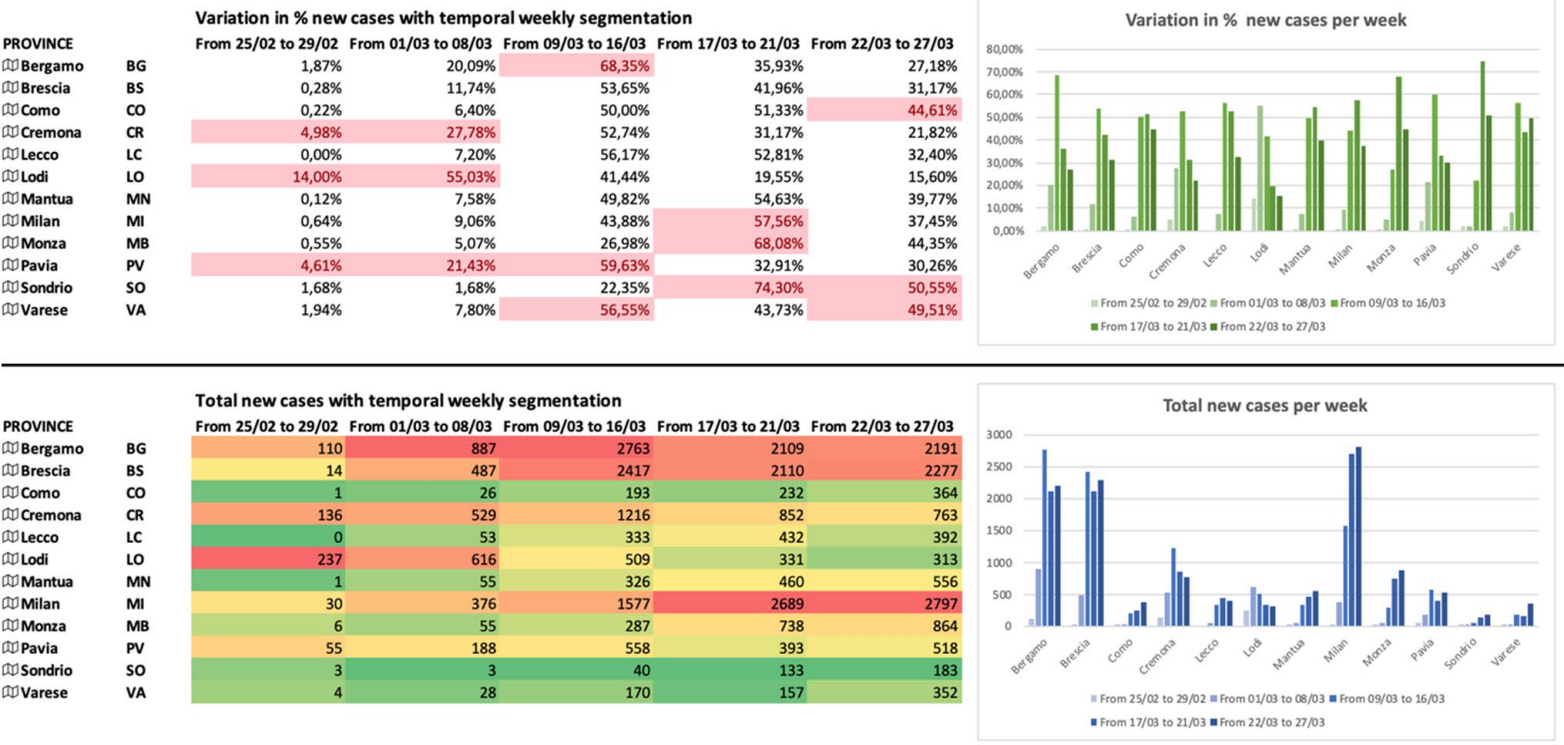
Weekly trend for new positive cases for all the Provinces in Lombardy Region (% variations vs. absolute values)

In Fig. 4, we report the evolution of the COVID-19 spread over time. We decided to group the new COVID-19-positive cases (reported daily by official agencies and media such as the web portal of Lombardy Region and the Department of Italian Civil Protection) by weeks. At the time of writing, we collected and grouped data from February 25 to March 27 into 5 groups. The figure shows the variation (%) and the total cumulative number of new positive cases, week by week. Numbers are also plotted in two histograms to show the evolution of epidemy in each Province. In the first week (from Feb. 02 to Feb. 29, 2020), the three main Provinces affected (in %) by COVID-19 are LO (the outbreak of COVID-19 in Italy), CR, PV, perfectly align with the outcomes coming from the O/D matrix of Fig. 2. However, if we consider the absolute values, the most infected Provinces are LO, CR, BG, and PV. In the second week (from March 01 to March 08, 2020), the three main Provinces affected (in %) by COVID-19 are again LO, CR, and PV with a significant increase (in %) for BG. As for absolute values, we observe a new strong increase for BS, and MI. The third week (from March 09 to March 16, 2020) is clearly the most critical week, with the explosion of BG, BS and a regression at LO. In the fourth week (from March 17 to March 21, 2020), we can observe the explosion of MI (already started the previous week) and MB, while we observe a small regression in BG and BS. The trend of COVID-19 positives is increasing in CO and LC too. In the last week (from March 22 to March 27, 2020), we can observe a critical variation (%) in CO, SO and VA, while for absolute values, the situation is still critical in MI, BG, BS, and MB. For MI and MB, the trend is increasing, while for BG and BS, the trend is decreasing. The graphical plot of these values clearly shows the trend of all the Provinces over time.

Furthermore, Figs. 2 and 4 can explain why MI and MB, which are geographically near to the outbreak LO, have been infected later than other Provinces, such as CR or PV: CR and PV are strongly connected with LO from a logistics point-of-view, while MI and MB have other preponderant logistics connections. Then, BS is strongly connected with CR and MN, thus, explaining the small delay in the COVID-19 spread in BS.

With this observation that a correlation exists between the goods and foods logistics and the diffusion of COVID-19, the outbreak at Codogno (LO) can be explained with the location of two of the most important and sizeable hubs for logistics in Italy and Europe.

This correlation can also explain why Lombardy Region has been so affected by the COVID-19 diffusion if compared to other big Italian Regions (such as Veneto Region that has 5 M inhabitants but only 9.000 positives to COVID-19, or Piemonte Region with 4.4 M inhabitants but only 8.900 total cases, at the time of writing.) and why the second infected region in Italy is Emilia Romagna (where foods and agriculture connections with Lombardy Region are so prevalent.)

Although Milan is close to Lodi, the trade in goods between the two cities is contained (the O/D matrix MI-LO mark only 1612 vehic./day): it could explain why in Milan the contagion has been delayed.

Milan instead exchanges a lot with Bergamo, Como and Varese. Perhaps, Milan has “received” the contagion from Bergamo and after that Milan may be “responsible” for the infection in Como, Varese and Monza.

This can be explained with the onion metaphor: the outbreak at Codogno (LO) is the center of the onion, and then the COVID-19 spread follows the onion layers in an irregular way. This unpredictable irregularity of each onion and of each layer, which compose an onion, can explain why the spread of the COVID-19 is not regular from a graphical point-of-view: the spread follows different rules than the geographical distance, as discussed in this paper. The different logistics interactions among the Provinces have a direct impact on the spread of COVID-19. LO was the center of the onion; PV and CR the first onion layer; BG and BS the second layer; MI, MN, LC, CO, VA the third layer, and at the end MB, SO. If we take a look to the most congested logistics connections, the secondary interactions (such as BG-BS, VA-MI, VA-CO) impacted on the second onion layer. This also explains why MB was the last affected Province: MB has its strong logistics connection with MI (9836 vehic./day) while a very low connection with LO (175 vehic./day). This means that MB was “protected” at the beginning by this weak connection with LO, but over time received the infection from MI.

Similar considerations may come from the O/D matrix about population intra/inter-travels for all the Provinces in Lombardy Region. It is clear that the main travel routes from LO are the following ones: LO–MI (49,218 travels), LO–CR (13,933 travels), and LO–PV (9792 travels). However, the connection LO–MI (from the MI point-of-view) is the sixth highest connection, after the connections: MI–MI, MI–MB, MI–VA, MI–PV, and MI–BG. The lowest number of travels is registered for the connection: LO–SO and MN–SO. In this case too, people travel can be considered as a good predictor of COVID-19 geographical spread.

## A Correlation Study on Decrees and Government Restrictions to Prevent the Diffusion of COVID-19

In this section, we report all the legislation and restrictions imposed over time by the Italian Government to prevent and reduce the spread of COVID-19. It is important to remember that the “patient 1” was detected in Codogno (Lodi) at the end of February (24th of February). Starting with the identification of the first COVID-19 outbreak in Italy and specifically in Lombardy Region, the Italian Government and the local regional government acted promptly to isolate the COVID-19 spread. Hereafter, we discuss each governmental measure separately, starting from the measures to prevent and contain the COVID-19 spread to the measures to support economy.

## Circulars and Restrictions to Prevent the Diffusion

January 22, 2020—Circulars of the Directorate General for Health Prevention of the Ministry of Health: containing indications on case management in healthcare facilities, the use of Personal Protective Equipment (PPE) for healthcare professionals and standard biosecurity precautions.

January 25, 2020—Order of the Minister of Health published in the Official Gazette—General Series, n. 21 of January 27, 2020: surveillance for those coming from infected areas.

January 30, 2020—Oder of the Minister of Health published in the Official Gazette—General Series, n. 26 of 1 February 2020: ban on flights from China.

January 31, 2020—Resolution of the Council of Ministers: for 6 months, it is declared a state of emergency on the national territory in relation to the health risk associated with the onset of diseases deriving from transmissible viral agents.

February 3, 2020—Order of the Head of the Civil Protection Department n. 630: it contains the “First urgent civil protection interventions in relation to the emergency linked to the associated health risk” with which the Head of the Civil Protection Department ensures the coordination of the necessary interventions.

February 21, 2020—Order of the Minister of Health:

1. The territorially competent health authorities are obliged to apply the quarantine measure with active surveillance, for 14 days, to individuals who have had close contacts with cases confirmed by COVID-19.
2. All individuals who, in the last 14 days, have entered Italy after having stayed in areas of China affected by the epidemic, as identified by the World Health Organization, are obliged to communicate this circumstance to the Prevention department of the health authority territorially competent.
3. Having acquired the communication referred to in paragraph 2, the territorially competent Health Authority implement the “fiduciary home stay measure” with active surveillance or alternative measures of equivalent efficacy, for people who arrived in Italy from epidemiological areas of risk or who develop diseases resulting from transmissible viral agents.

## Decrees to Contain the Diffusion and Start to Support the Economy

February 23, 2020: Decree nr. 6—the red zone

Urgent measures to avoid the spread of COVID-19. In municipalities or areas “red”, it is decreed:

1. the prohibition of any person presents in the municipality or area from leaving the municipality or area concerned;
2. prohibition of access to the municipality or area concerned;

February 23, 2020: The Minister of Health, with the President of the Lombardy Region—the yellow zone

In all Lombard municipalities or areas different from the red zone, it is decreed:

1. suspension of events and of any form of meeting in a public or private place;
2. closure of schools and universities—only e-learning activities are granted—, excluding specialists and trainees of the health professions;
3. suspension of the opening services to the public (museums, cinemas, etc.)
4. suspension of all study trips, both nationally and abroad;
5. fiduciary home stay measure with active surveillance for people who arrived in Italy from epidemiological areas of risk, identified by the World Health Organization;
6. suspension of bankruptcy proceedings.
7. Commercial activities are available in these terms:
  bars, pubs and other entertainment open to the public are closed from 18.00 to 6.00; measures will be defined to avoid gatherings in these premises;
  shops located within shopping centers and markets, are closed on Saturdays and Sundays;
  trade fairs are closed;
  merchants are invited to ensure suitable precautionary measures.

February 24, 2020: Suspension of the deadlines for the fulfillment of tax obligations in favor of taxpayers affected by the epidemiological emergency caused by COVID-19 (20A01299) (Official Gazette—General Series n.48 of 26-02-2020).

February 29, 2020: Measures to support families, workers and businesses in the red zone and other people particularly affected by the emergency such as tour operators.

March 1, 2020 Decree: Implementation of the decree 23 February 2020 n. 6 containing urgent measures regarding the containment and management of the epidemiological emergency from COVID-19 (20A01381) (Official Gazette—General Series n.52 of 01-03-2020).

In the yellow zone, opening of bars, pubs, restaurants, museums etc. is possible but conditional on the safety distance between the people.

In Lombardy Region and in the province of Piacenza, the activities of gyms, sports centers, swimming pools, swimming centers, wellness centers, spas, cultural centers, social centers, and leisure centers continue to be suspended.

## Decrees to Manage the COVID-19 Emergency

March 4, 2020 Decree: Implementation of the decree 23 February 2020 n. 6 containing urgent measures regarding the containment and management of the epidemiological emergency from COVID-19 (20A01475) (Official Gazette—General Series n. 55 of 04-03-2020).

Many measures of the decree 23 February 2020 for the yellow zone are applied all throughout the country. The schools are closed in the red zone. In the rest of country, the lessons are suspended but teachers and employees can work at school.

March 8, 2020 Decree: the orange zone, the leak and the exodus from north to south

Implementation of the decree 23 February 2020 n. 6 containing urgent measures regarding the containment and management of the epidemiological emergency from COVID-19 (20A01522) (Official Gazette—General Series n.59 of 08-03-2020).

Urgent measures to contain the contagion in the Lombardy Region and in the provinces of Modena, Parma, Piacenza, Reggio nell'Emilia, Rimini, Pesaro and Urbino, Alessandria, Asti, Novara, Verbano-Cusio-Ossola, Vercelli, Padua, Treviso, Venice.

The publication of a draft of the decree triggers an exodus from north to south of the students.

March 9, 2020 Decree: Implementation of the decree 23 February 2020 n. 6 containing urgent measures regarding the containment and management of the epidemiological emergency from COVID-19 (20A01558) (Official Gazette—General Series n.62 of 09-03-2020).

The sporting events and competitions of each order and discipline are suspended.

March 11, 2020 Decree: Implementation of the decree 23 February 2020 n. 6 containing urgent measures regarding the containment and management of the epidemiological emergency from COVID-19 (20A01605) (Official Gazette—General Series n.64 of 11-03-2020).

Italy is all orange zone.

March 22, 2020 Decree: Implementation of the decree 23 February 2020 n. 6 containing urgent measures regarding the containment and management of the epidemiological emergency from COVID-19 (20A01807) (Official Gazette—General Series n.76 of 22-03-2020).

All unnecessary industrial and commercial production activities must be suspended by 25 March.

Italy is all red zone.

**Fig. 5:**
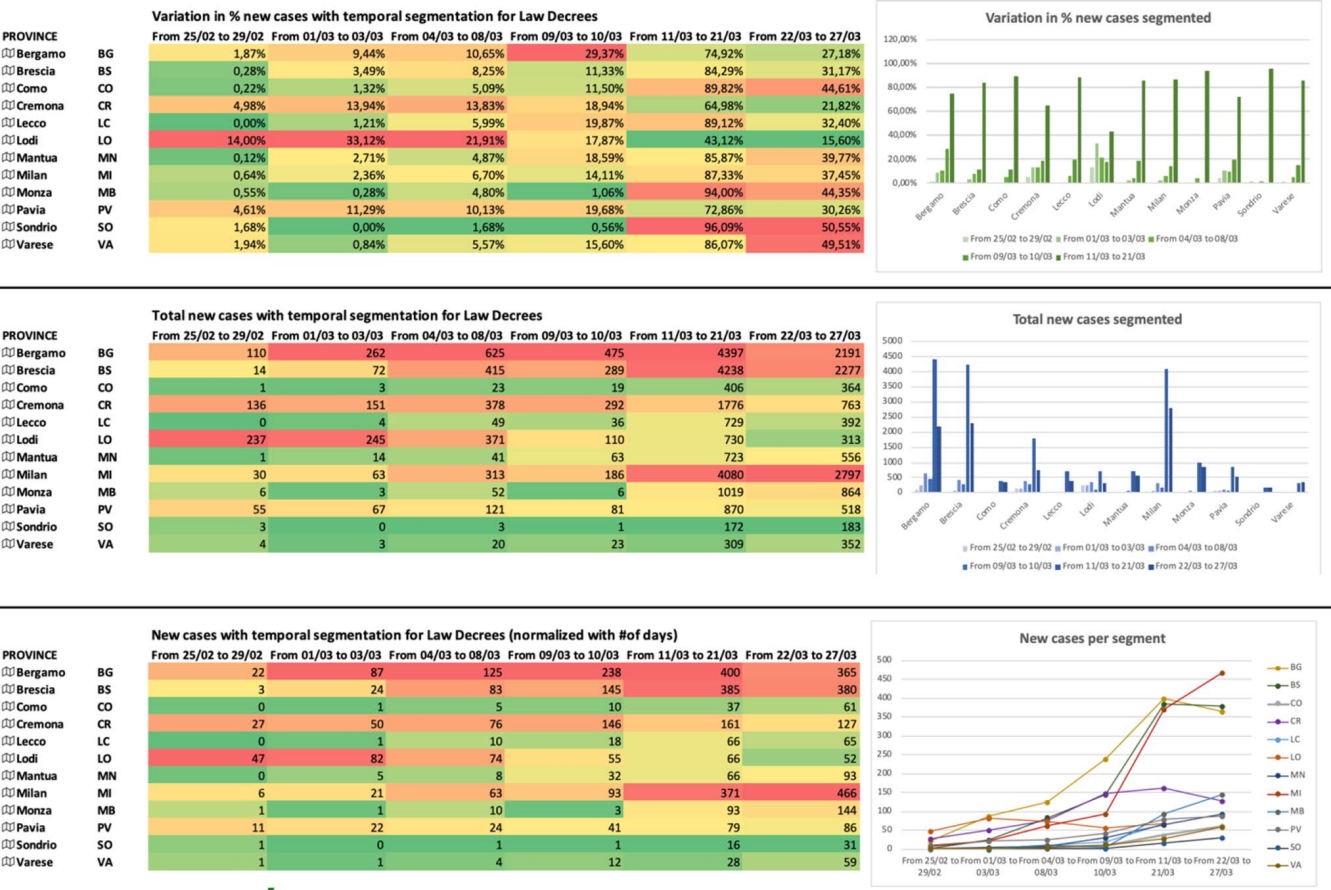
Trend segmented by Italian Decrees for new positive cases for all the Provinces in Lombardy Region (% variations vs. absolute values vs. normalized values)

In Fig. 5, we report the evolution of the COVID-19 spread, over time. We decided to group the new COVID-19-positive cases (reported daily by official agencies and media such as the web portal of Lombardy Region and the Department of Italian Civil Protection) by following the dates of signature of each piece of legislation to manage the emergency. At the time of writing, we collected and grouped data from February 25 to March 27 into 6 groups. The figure shows the variation (%), the total cumulative number of new positive cases, and the normalized number of new cases, segmented by dates of Legislation. Numbers are also plotted in three histograms to show the evolution of epidemy in each Province of Lombardy Region. The three tables are colored with a set of colors ranging from green (low variations or concentrations) to red (high variations or concentrations). For example, in the first segment (from Feb. 25 to Feb. 29), LO is the Province with the highest variation of 14.00% (since LO was the COVID-19 outbreak in Italy), while LC is the Province with the lowest variation of 0.00%. In the second segment (from March 01 to March 03), LO has the highest variation (33.12%), while BG the highest number of new cases (262). In the third segment (from March 04 to March 08), LO, CR, PV, and BG show the highest variations, and also the absolute numbers are impressive. It is clear that, right now, the restrictions imposed by the Decree nr.6 (red zone for Codogno) have not a real impact yet: in fact, all the indicators of Fig. 5 are still increasing, thus, indicating a continuous increase in the COVID-19 spread. In the fourth segment (from March 09 to March 10), we observe the first real impact of the Decree nr.6 (red zone for Codogno): LO is decreasing its % variation, and also the absolute number of new COVID-19 cases is slowing down. It is important to remember that the first adopted restrictions in LO were very stringent (red zone). CR is the only other Province that clearly shows a positive trend (i.e., with positive we mean a reduction in the number of new COVID-19 cases).

**Fig. 6:**
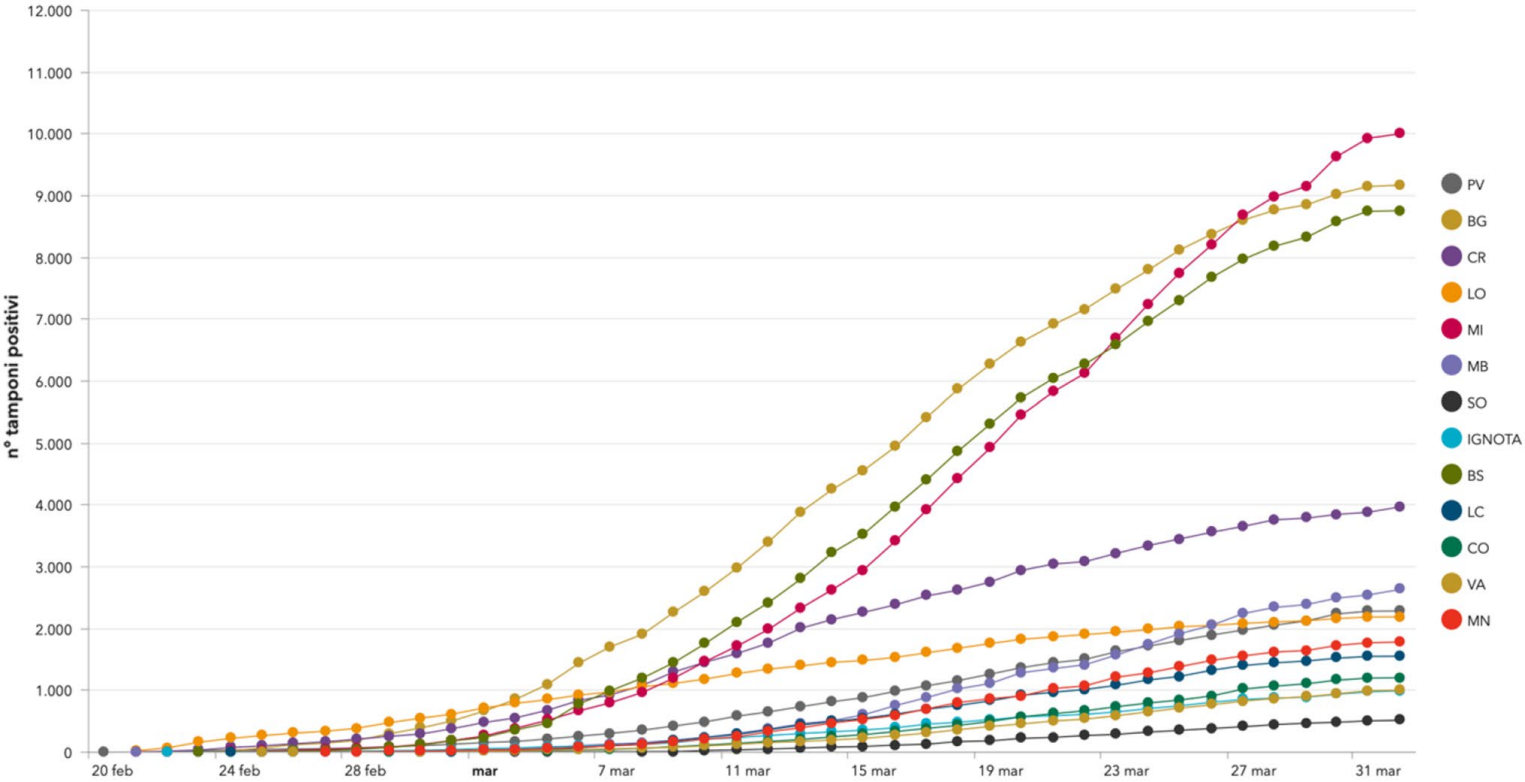
Cumulative curves for total COVID-19 cases per each Province in Lombardy Region ( source: Lombardy Region Web Portal)

Unfortunately, for all the other Decrees, we do not have a clear understanding yet on their effect on the trend of variations and absolute numbers, at the time of writing. However, it is important to highlight that all the curves for the cumulative number of COVID-19 cases for each Province are not exponential functions (as for typical epidemies) but less steep (they are power functions) as shown in Fig. 6 [9].

## Discussion

Additional informal considerations may raise from these preliminary data elaborations.

Surveillance for those coming from infected areas from 22nd January, and ban on flights from China from 30th January has not halted the infection in Italy.

Moreover, the Champions League match Atalanta–Valencia was played on 19th February in Milan, 4 days before the decree of 23rd February containing urgent measures to avoid the spread of COVID-19. The contagion curve in Bergamo starts to rise 10 days later, and 14 days after the match the contagion curve is higher in Bergamo than in Lodi, growing especially in Alzano Lombardo, where there is a hospital, and in Nembro.

The contagion curve in Milan starts to rise 12 days after the match Atalanta–Valencia, but other factors more likely to have had influence, included the flows of interchange between Milan and Bergamo the days after the match: indeed, on the day of the match, the Milan underground was full of Spanish fans more so than Atalanta’s fans because from Bergamo to San Siro Stadium it is easier for locals to go by car or private bus than by public transport.

The legislated containment measures of 23rd February were contested immediately, and further legislation on 1st March introduced a slight reduction in restrictions for the yellow zone, which also included Milan and Bergamo. Until the legislation of 8th March, people flocked to parks, bars and restaurants, did not respect social distancing safety limits and did not wear masks. The contagion curve of Milan, Bergamo and Brescia, that was already growing, continued to grow quickly between 11th and 21st March. It is anticipated that other Lombardy provinces with an active nightlife will register different trend.

The publication of draft legislation triggered an exodus from North-Italy to South-Italy of students between the 7th and 8th March: people who arrived in the south were requested to notify their move to the authorities and to respect fiduciary home stay measures. Probably thanks to this, the impact of the exodus has been statistically satisfactory.

Legislative measures have reduced the contagion significantly, but are not enough to completely explain the spread of the contagion, and needs to be considered in conjunction with other factors such as the flows of interchange between territories, the outbreaks that occurred in hospitals, the events, individual behaviors and their social habits.

Many observers are theorizing that in democratic countries, it is more difficult to adopt efficient legislative measures during a pandemic, but it is important to recognize that many factors that have had an impact on the spread of the contagion do not necessarily depend on the degree of democracy of a country, and that in reality the curve of Italy is similar to the curve of China.

## Conclusion

From these analyses, the relationship between how the virus has spread and how the goods and food transport is distributed on the Lombard territory were highlighted. Based on these correlations and evidence, is reasonable to explain why Codogno was the first outbreak in Italy: in fact, in the south of Lodi are located the biggest logistics and distribution hubs in Italy and among the largest in Europe. It is clear that this does not mean that the vehicle of contagion of the COVID-19 is goods and food, but rather the intra-personal relationships that the distribution of the goods (food and non-food) creates on the Lombard territory.

Moreover, this study highlights the empirical correlation between the geographical spread of the infection and the movements of the inhabitants on the territory.

Statistical/mathematical correlation studies that prove these empirical observations, coupled to big data studies, could allow to verify the real correlation between the dependent variable (contagions) and the independent variables (logistics transport + citizens travel) to generalize these models able to forecast the trends of contagion evolution, well in advance and on the whole national territory.
